# Lipoprotein Proteomics and Aortic Valve Transcriptomics Identify Biological Pathways Linking Lipoprotein(a) Levels to Aortic Stenosis

**DOI:** 10.3390/metabo11070459

**Published:** 2021-07-16

**Authors:** Raphaëlle Bourgeois, Jérôme Bourgault, Audrey-Anne Despres, Nicolas Perrot, Jakie Guertin, Arnaud Girard, Patricia L. Mitchell, Clarisse Gotti, Sylvie Bourassa, Corey A. Scipione, Nathalie Gaudreault, Michael B. Boffa, Marlys L. Koschinsky, Philippe Pibarot, Arnaud Droit, Sébastien Thériault, Patrick Mathieu, Yohan Bossé, Benoit J. Arsenault

**Affiliations:** 1 Centre de Recherche de l’Institut Universitaire de Cardiologie et de Pneumologie de Québec, Québec, QC G1V 4G5, Canada; raphaelle.bourgeois@criucpq.ulaval.ca (R.B.); jerome.bourgault@criucpq.ulaval.ca (J.B.); audreyannedespres@gmail.com (A.-A.D.); nicolas.perrot@criucpq.ulaval.ca (N.P.); jakie.guertin@criucpq.ulaval.ca (J.G.); arnaud.girard@criucpq.ulaval.ca (A.G.); patricia.mitchell@criucpq.ulaval.ca (P.L.M.); nathalie.gaudreault@criucpq.ulaval.ca (N.G.); Philippe.Pibarot@med.ulaval.ca (P.P.); sebastien.theriault@criucpq.ulaval.ca (S.T.); patrick.mathieu@fmed.ulaval.ca (P.M.); yohan.bosse@criucpq.ulaval.ca (Y.B.); 2 Department of Medicine, Faculty of Medicine, Université Laval, Québec, QC G1V 0A6, Canada; 3 Proteomics Platform of the CHU de Québec, QC G1V 4G2, Canada; clarisse.gotti@crchudequebec.ulaval.ca (C.G.); sylvie.bourassa@crchudequebec.ulaval.ca (S.B.); arnaud.droit@fmed.ulaval.ca (A.D.); 4 Toronto General Research Institute, University Health Network, Toronto, ON M5G 2C4, Canada; Corey.Scipione@uhnresearch.ca; 5 Robarts Research Institute, London, ON N6A 5B7, Canada; mboffa@uwo.ca (M.B.B.); mlk@robarts.ca (M.L.K.); 6 Centre de Recherche du CHU de Québec, Québec, QC G1V 4G2, Canada; 7 Department of Molecular Biology, Medical Biochemistry and Pathology, Faculty of Medicine, Université Laval, Québec, QC G1V 0A6, Canada; 8 Department of Surgery, Faculty of Medicine, Université Laval, Québec, QC G1V 0A6, Canada; 9 Department of Molecular Medicine, Faculty of Medicine, Université Laval, Québec, QC G1V 0A6, Canada

**Keywords:** lipoprotein(a), calcific aortic valve stenosis, aortic valve, proteomics, transcriptomics

## Abstract

Lipoprotein(a) (Lp(a)) is one of the most important risk factors for the development of calcific aortic valve stenosis (CAVS). However, the mechanisms through which Lp(a) causes CAVS are currently unknown. Our objectives were to characterize the Lp(a) proteome and to identify proteins that may be differentially associated with Lp(a) in patients with versus without CAVS. Our second objective was to identify genes that may be differentially regulated by exposure to high versus low Lp(a) levels in explanted aortic valves from patients with CAVS. We isolated Lp(a) from the blood of 21 patients with CAVS and 22 volunteers and performed untargeted label-free analysis of the Lp(a) proteome. We also investigated the transcriptomic signature of calcified aortic valves from patients who underwent aortic valve replacement with high versus low Lp(a) levels (*n* = 118). Proteins involved in the protein activation cascade, platelet degranulation, leukocyte migration, and response to wounding may be associated with Lp(a) depending on CAVS status. The transcriptomic analysis identified genes involved in cardiac aging, chondrocyte development, and inflammation as potentially influenced by Lp(a). Our multi-omic analyses identified biological pathways through which Lp(a) may cause CAVS, as well as key molecular events that could be triggered by Lp(a) in CAVS development.

## 1. Introduction

Calcific aortic valve stenosis (CAVS) is the most prevalent valvulopathy worldwide, affecting approximately 2% of the population over 65 years old [1,2]. Its prevalence is increasing with population aging [3]. CAVS results from an active inflammatory process, including lipoprotein infiltration and oxidation, immune cell activation and infiltration into the valve leaflets cells, and osteoblastic transition of valvular interstitial cells (VICs) leading to fibrocalcific remodelling [4,5,6]. CAVS shares many of the risk factors for atherosclerotic cardiovascular diseases (ACVD), such as age, male sex, hypertension, higher low-density lipoprotein (LDL) cholesterol, type 2 diabetes, tobacco use, and elevated lipoprotein(a) (Lp(a)) levels [7,8,9,10,11,12]. However, there is currently no specific pharmacological treatment for the prevention or treatment of CAVS. The only treatment for symptomatic end-stage process disease patients is surgical or transcatheter valve replacement [6].

Many studies sought to identify potential new biomarkers or therapeutic targets for CAVS using different “omic” approaches, such as plasma [13,14,15], heart valve tissue proteomics [16,17,18], transcriptomic [19,20], or genome-wide analysis [11,21,22]. In genome-wide analysis, *LPA* was identified as one of the main risk factors for the development of aortic valve calcification and CAVS. Lp(a) is an LDL-like particle whereby the highly polymorphic apolipoprotein(a) (encoded by *LPA* on chromosome 6) is linked to apolipoprotein (apo) B via a disulfide bridge [23]. Apo(a) is composed of different kringle domains (KIV-1 to KIV-10 and KV), all present once, except KIV-2, which can be repeated several times [24]. The copy number of KIV-2 is inversely correlated with Lp(a) concentration and with the risk of ACVD [25]. Lp(a) not only possesses the atherogenic properties of LDL, but it was found to be the preferential carrier of proinflammatory oxidized phospholipids [26] (OxPL). OxPLs have an important role in the initiation and progression of ACVD or CAVS [27], and have been found colocalized with Lp(a) in aortic lesions [28,29,30]. Moreover, it was found that high levels of OxPL and Lp(a) may be independently associated with CAVS progression [31].

In addition to apoB and apo(a), Lp(a) might transport other proteins in the bloodstream. A study identified autotaxin (ATX), a lysophospholipase D which converts lysophosphatidylcholine into lysophosphatidic acid, to be transported by Lp(a) [32]. High Lp(a), combined with high ATX levels, were associated with a faster progression of CAVS [33] in patients with coronary artery disease. Analyses of Lp(a) and LDL proteomes have also identified a potential role of Lp(a) in wound healing and complement activation [34]. In a comparative analysis of the Lp(a) and LDL proteomes isolated from the same individuals, we also recently reported profound differences between these two fractions, with Lp(a) carrying many more proteins that LDLs [35]. It is, however, unknown if the proteome of Lp(a) differs across disease states such as CAVS.

Ex vivo, incubation of human valvular endothelial or interstitial cells with Lp(a) induces the expression of osteogenic genes, such as *IL6*, *BMP2*, and *RUNX2* [28,29]. This effect may be dependent on OxPLs. To our knowledge, there have been no studies using unbiased transcriptomic approaches to identify the mechanisms through which Lp(a) may cause CAVS.

Multi-omic approaches are increasingly used in basic cardiovascular sciences to optimize risk prediction or patient stratification and to identify new pathobiological mechanisms leading to ACVD or CAVS. Since the majority of people with high Lp(a) levels will not develop CAVS, our first objective was to identify the potential differences in the Lp(a) proteome between individuals with versus without CAVS. Our second objective was to obtain new insight on the mechanisms or pathways through which Lp(a) might cause aortic valve calcification in an unbiased manner. We therefore used a transcriptomic approach on explanted calcified aortic valves, and compared gene expression levels in patients with high versus low Lp(a) levels.

## 2. Results

### 2.1. Proteomic Analysis of Lp(a) Proteome from Patients with Versus without CAVS

The label-free analysis of the Lp(a) proteome identified 172 proteins associated with Lp(a) (regardless of CAVS status). These are listed in order of intensity in Table S1. Next, we compared the Lp(a) proteome of patients with CAVS to that of controls with an anatomically normal aortic valve. Participants’ characteristics are presented in Table 1.

A total of 13 proteins were differently associated with the Lp(a) of participants with versus without CAVS, with a *p*-value < 0.05. All the fold changes were found above 1.7. Among these 13 proteins, 9 of them were found to be preferentially associated with the Lp(a) of patients with CAVS: LCAT, NCAM1, VASN, PON3, SerpinG1, LAMP2, MCAM, MADCAM1, and CHL1. Conversely, the four other proteins were found to be preferentially associated with the Lp(a) of controls: beta-2-glycoprotein 1 (β2GPI), IGHM, IGKV2D-30, and JCHAIN;IGJ (Table 2).

**Figure 1:**
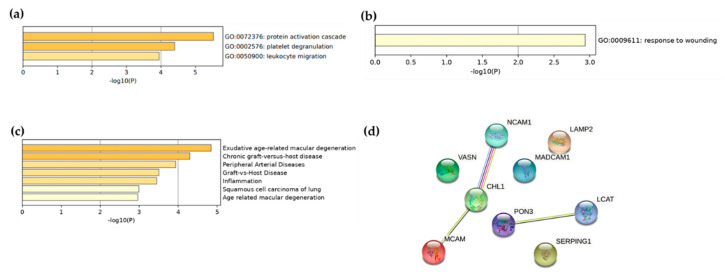
Enrichment analysis of proteins found to be associated with Lp(a) regardless of (**a**) CAVS status and (**b**) in CAVS only. (**c**) DisGeNET enrichment analysis of proteins found to be associated with CAVS Lp(a) and (**d**) interaction analysis between the proteins identified as preferentially associated with the CAVS Lp(a) by STRING-db.

Enrichment pathway analysis performed with Metascape [36] revealed that the 13 proteins identified were mainly involved in protein activation cascade, platelet degranulation, and leukocytes migration pathways (Figure 1a). When performing pathway analyses with the nine proteins associated with the CAVS Lp(a), we found one enriched pathway: response to wounding (Figure 1b). Moreover, a DisGeNET [37] analysis of these nine proteins found them to be associated with several diseases, such as peripheral arterial disease and inflammation (Figure 1c). Finally, using STRING-db, we observed that MCAM (co-expression) and NCAM1 (co-expression, protein homology, and interaction demonstrated experimentally between putative homologs) are known to interact with CHL1, and PON3 with LCAT (co-expression of putative homologs in other organisms) (Figure 1d).

### 2.2. Transcriptomic Analysis of Explanted Calcified Valves from Patients with CAVS

**Figure 2:**
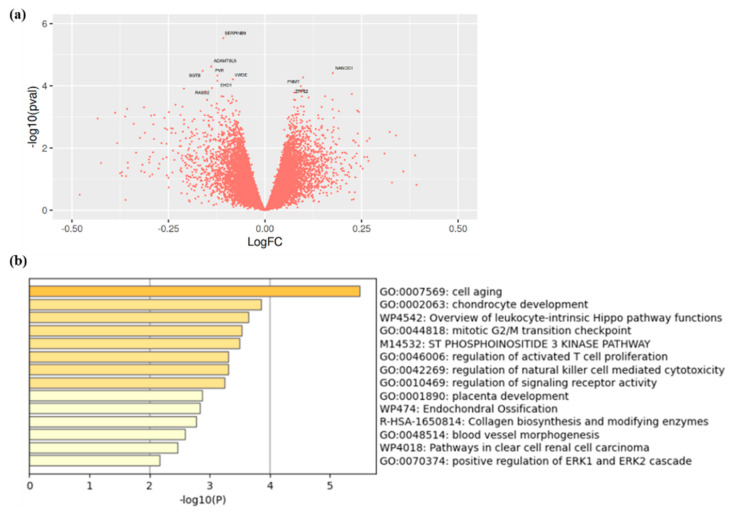
(**a**) Volcano plot showing the genes identified in calcified valve of patients with high versus low Lp(a) and (**b**) enrichment analysis of genes found to be the most influenced by Lp(a) concentrations in explanted calcified aortic valves of patients with high versus low Lp(a).

We compared the gene expression signature of calcified aortic valves from CAVS patients with high vs. low Lp(a) levels. Participants were matched for age, sex, tobacco, and statin use. Participant characteristics are shown in Table 3. Lp(a) levels were 29.2 ± 28.6 nmol/L and 217.6 ± 93.1 nmol/L in the low and high Lp(a) group, respectively. Transcriptomic analysis revealed that one gene, SERPINB9, reached an arbitrary threshold of statistical significance (*p* < 1.0 × 10^−5^) and may be downregulated in aortic valve tissue from participants with high Lp(a) (Figure 2a and Table S2). However, after FDR correction, no gene passed the statistical significance threshold (*p*-value for SERPINB9 was 2.94 × 10^−6^, and 0.070 after FDR correction). We then performed an enrichment pathway analysis on the 100 genes most influenced by Lp(a) levels. Interestingly, among all the pathways identified with Metascape, the most enriched pathways involved cell aging, chondrocyte development, and inflammation (Figure 2b).

## 3. Discussion

The role of Lp(a) in the development of CAVS is well established. However, in light of the fact that only a minority of people with high Lp(a) levels will develop CAVS, we investigated the potential difference of the Lp(a) proteome in patients with versus without CAVS. Our untargeted proteomic approach identified 172 proteins linked to Lp(a) in the bloodstream and identified nine proteins (LCAT, NCAM1, VASN, PON3, SerpinG1, LAMP2, MCAM, MADCAM1, and CHL1) that may be preferentially associated with the Lp(a) of patients with CAVS. Four proteins (β2GPI, IGHM, IGKV2D-30, and JCHAIN;IGJ) may be found in lower concentrations in the Lp(a) of patients with CAVS. These proteins are involved in several key atherogenic pathways, including protein activation cascade, platelet degranulation, leukocyte migration, and response to wound healing. We also investigated the transcriptome of explanted calcified valves of patients with CAVS with either high or low Lp(a) levels to identify genes and pathways that could be differentially regulated according to Lp(a) levels, and found that exposure to higher Lp(a) levels may be associated with the downregulation of the *SERPINB9* gene as well as the dysregulation of genes involved in cell aging, chondrocyte development, and inflammation.

Most of the proteins that were found to be more present on the Lp(a) isolated from patients with CAVS compared to controls appeared to be involved in cell adhesion, immune cell recruitment, and migration, which may be important mechanisms in the progression of CAVS. For instance, vasorin (VASN) was found to modulate arterial response to injury in vivo [38]. It also appears to be involved in cellular migration and proliferation/differentiation [39]. Neural cell adhesion molecule-1 (NCAM1) is involved in cell–cell and matrix adhesion in cardiomyocytes adhesion [40], and is upregulated under metabolic stress [41]. Melanoma cell-adhesion molecule (MCAM) is an adhesion molecule from the immunoglobulin superfamily involved in monocyte transmigration [42,43] and angiogenesis. Its short isoform also produces proinflammatory cytokines [44]. It was reported that MCAM could associate with the monocytes and promote interaction with activated endothelial cells causing atherosclerosis progression [43]. MCAM upregulation also mediates oxidized lipids uptake in macrophages, contributing to macrophage retention in atheroma [45]. Another protein that was found to be higher in the Lp(a) isolated from patients with CAVS compared to controls is mucosal vascular addressin cell adhesion molecule 1 (MADCAM1). This protein is expressed in cardiomyocytes and endothelial cells in vitro and could induce interaction of endothelial cells with leukocytes [46]. Cell adhesion molecule close homolog of L1 (CHL1) has an important role in the development and regeneration of the nervous system [47,48], and is linked to pathologies, such as mental retardation [49] or schizophrenia [50]. Lysosomal-associated membrane protein 2 (LAMP2) is mainly found on the surface of lysosomes, where its role is to maintain lysosomal membrane integrity, lysosome biogenesis, and lysosome fusion with autophagosome in macro autophagy [51]. Recently, it was suggested that LAMP2 was involved in surface expression of RANKL of osteoblast, thus participating in osteoclastogenesis, a process found in aortic valve calcification [52]. LAMP2 deficiency is associated with Danon disease, which features cardiomyopathy, myopathy, and mental retardation [53]. SerpinG1, a protease inhibitor, might help reduce monocyte activation and intimal hyperplasia [54]. It can inactivate several fibrinolytic and coagulation system proteases, making this protein a protector against atherosclerosis, counteracting the role of Lp(a) in the development and progress of atherosclerosis, and so, potentially, CAVS. Interestingly, SerpinG1 was found in control and diseased aortic valves, with decreased levels in the diseased ones [55]. Vasorin and SerpinG1, which were found to be more abundant in the Lp(a) of CAVS patients compared to controls in this study, were already identified in studies investigating the blood proteome of patients with CAVS [13,14]. Initiation of aortic stenosis involves endothelial dysfunction, which is amplified in the context of exposure to elevated levels of apoB-containing lipoproteins. Two of the proteins we identified, LCAT and PON3, are involved in lipoprotein metabolism and modification. The role of LCAT in cardiovascular diseases and atherosclerosis is still controversial, as LCAT deficiency is common in several chronic disorders [56], whereas some studies suggest that an increase in LCAT activity is associated with an increased formation of small and dense LDL [57,58], which are more atherogenic and prone to oxidation. PON3, on the other hand, may prevent oxidative modification of LDL in atherosclerosis by degrading lipoperoxides in lipoproteins [59,60].

We identified four proteins that may be more abundant in the Lp(a) of participants without CAVS, three of them being associated with the immunoglobulin family (IgM, JCHAIN, IGKV-2D-30) and β2GPI. β2GPI activates lipoprotein lipase and triglyceride-rich lipoprotein (TRL) catabolism [61]. High TRL levels have recently been linked with CAVS [62]. β2GPI was found to associate with ox-LDL [63] and, more interestingly, to Lp(a) particles [64]. As this protein also has a role in clearing the non-self particles [65], it was proposed that Lp(a) could be taken up via β2GPI recognition mechanism by macrophages [66]. Mutations in *APOH* (the gene encoding β2GPI) are also associated with an impaired association of β2GPI with phospholipids [67] and with LDL particle size [68]. Although this would require experimental confirmation, one can hypothesize that the Lp(a) of control subjects might be cleared by a β2GPI-mediated mechanism to a greater extent than the CAVS Lp(a) and/or have a potentially lower OxPL content. Interestingly, a novel variant at the *APOH* locus was recently found to be associated with increased Lp(a) levels [69]. Finally, IGHM is involved in primary defense mechanisms and has a wide variety of properties, allowing it to participate in different pathophysiologies, such as infection, inflammation, or atherosclerosis [70]. Clinical studies have also shown an inverse relationship between cardiovascular events and specific IGHM [71,72]. IGHM also facilitates apoptotic cell clearance, which could promote inflammation resolution. Interestingly, antigenic targets present in atherosclerotic plaques as ox-LDL are bound to natural IGHM [73,74].

Our transcriptomic approach identified *SERPINB9* as being potentially expressed at lower levels in the valves of patients with high Lp(a) compared to those with lower Lp(a) levels. SerpinB9 is an intracellular inhibitor of granzyme B activity, and was discovered in T-cells to protect them from apoptosis [75]. Granzyme B is a serine protease family member which induces cell death by different mechanisms, and is also associated with extracellular matrix degradation [75]. Granzyme B expression was found to be 7-fold higher in calcified aortic valves, compared to aortic valves explanted from patients without CAVS [76]. It was demonstrated that this protein could be reduced in human atherosclerotic lesions [77]. Altogether, this suggests that people with less Lp(a) could be more protected from granzyme B apoptosis by a higher level of SerpinB9 expression, thus indicating a potentially different mechanism in the presence of lifelong exposure to high Lp(a) levels. A pathway analysis of the top genes most influenced by Lp(a) levels revealed that the main pathway identified appeared to involve dysregulation of genes encoding cell aging, chondrocyte development, and inflammatory pathways. These findings support the notion that plasma Lp(a) levels may be a key determinant of healthy aging, as supported by studies showing a strong association between Lp(a) levels and parental lifespan, health span, and all-cause mortality [78,79,80,81]. Pathways linking Lp(a) with cell aging may involve cellular senescence and cell cycle inhibition via the PI3K/AKT/mTOR pathway. Chondrocytes produce and maintain extracellular matrix collagens and proteoglycans, and may eventually differentiate into osteoblasts, which is a key molecular event in the development of aortic valve calcification. These results, therefore, support the notion that Lp(a) levels are robustly associated with macro- and microcalcification [11,82]. Finally, our transcriptomic analysis revealed several inflammatory pathways that may be influenced by lifelong exposure to high Lp(a) levels, including T lymphocyte proliferation and natural killer-cell-mediated lipotoxicity. These results further extend the results of the proteomic study by suggesting that several inflammatory pathways may be influenced by Lp(a) in the context of CAVS.

To our knowledge, our study may be the first study to compare the Lp(a) proteome across disease states and the first comparing the Lp(a) proteome in patients with versus without CAVS. We also could not find another study comparing the aortic valve transcriptomic response to lifelong exposure to high Lp(a) levels in such a large dataset. Limiting our findings however is the fact that, although our analysis could identify several new proteins that could be transported by Lp(a), this analysis was semiquantitative. Additional studies should be performed to confirm these results and to measure the proteins we identified in a quantitative manner. Furthermore, the lack of replication studies either in our proteomic and in our transcriptomic study is another limitation of this work. However, we were the first to perform label-free analysis of isolated Lp(a) fractions of 43 participants, which is the biggest Lp(a) sample cohort to date.

## 4. Materials and Methods

### 4.1. Study Participants

We recruited 21 participants with high Lp(a) levels (>125 nmol/L) and mild to severe CAVS at the echocardiography laboratory of the Quebec Heart and Lung Institute. Exclusion criteria included mitral valve stenosis, mitral or aortic insufficiency (moderate or more), heart failure (ejection fraction < 40%), CAVS from rheumatic etiology or cancer requiring radiotherapy in the thoracic area before CAVS diagnosis. Pregnant or lactating women were also excluded from the study. We also recruited 22 controls without CAVS and with high Lp(a) levels (>125 nmol/L) through advertisements. The study protocol was approved by the Ethics Committee of the Quebec Heart and Lung Institute, and all patients signed a written informed consent. Each participant completed detailed questionnaires, as previously described [82]. Plasma Lp(a) levels were measured using a turbidimetric assay, Tina-quant lipoprotein(a) Gen.2 system (Cobas integra 400/800, Roche Diagnostics, Mannheim, Germany).

### 4.2. Lipoprotein Isolation

Lp(a) subfractions were isolated from these 43 participants, as previously described [35,83,84]. Briefly, fresh serum was ultracentrifugated with a gradient of iodixanol solution (Optiprep). Three subfractions were identified after ultracentrifugation and the fraction enriched with Lp(a), as assessed with Sebia Hydragel LIPO + Lp(a) (Sebia, Lisses, France), was submitted to a combination of size exclusion and affinity chromatography. Lp(a) fraction purity was then assessed with Sebia Hydragel LIPO + Lp(a) to ensure no meaningful amounts of LDL or HDL were found with Lp(a).

### 4.3. Assessment of Lp(a) Proteome by Nanolc-MS/MS

The proteome of Lp(a) was evaluated as previously described [35]. Briefly, proteins from Lp(a) samples were precipitated overnight with acetone at −20 °C, then resuspended, reduced, alkylated, and, finally, digested with trypsin. An equivalent of 2 μg of each sample was then injected into a Dionex UltiMate 3000 nanoRSLC chromatography system (Thermo Fisher Scientific, Waltham, MA, USA), coupled with a nanoelectrospray source to an Orbitrap Fusion Tribrid mass spectrometer (Thermo Fisher Scientific). The acquired spectra were searched against the Uniprot complete proteome homo sapiens database (21 April 2019, 74,435 entries) using the Andromeda search engine in MaxQuant software v. 1.6.7.0 (Max Planck Institute of Biochemistry, Martinsried, Germany). Intensity values were first normalised by applying a normalization factor calculated from the median of all protein intensities of each sample. Missing values were then imputed with a noise value corresponding to the first percentile of all protein intensities. A protein was considered to be quantifiable only if it possessed at least 75% replicate values (16 of 21 samples in CAVS or 17 of 22 samples in controls) in one group with at least 2 peptides per protein. Statistical analyses were conducted on a mean CAVS Lp(a) intensity/mean control Lp(a) intensity ratio with Limma *t*-test). A multiple-test-corrected *p*-value adjusting FDR (set at 1% in MaxQuant) below 0.05 was considered significant. Data analysis was conducted with RStudio 1.1.383.

### 4.4. Transcriptomic Analysis of Explanted Calcified Valves from Patients with CAVS

Calcified explanted valves were recovered from the Quebec Heart and Lung Institute biobank from 240 subjects who underwent aortic valve replacement. We included all study participants with an Lp(a) level > 125 nmol/L and matched them on a 1:1 ratio with participants with the lowest Lp(a) levels according to age, sex, tobacco use, and statin use. The study protocol was approved by the Ethics Committee of the Quebec Heart and Lung Institute, and all patients signed a written informed consent. We obtained two groups of 59 participants. RNA was extracted from explanted valves leaflets using RNeasy Plus Universal Mini Kit (Qiagen). Gene expression was evaluated using the Illumina HumanHT-12 v4 Expression BeadChip. Standard microarray processing and quality control analysis were performed as previously described [22,85]. Briefly, the raw data were quantile normalized after log2-transformation with the lumi package in R. Probe sequences were mapped to RefSeq B38, GENCODE v24 B38, mRNA B38, and the human genome (GRCh B38) using Bowtie, and probes not mapping to any coding region were removed, leaving a total of 45,699 probes. Robust fitting of linear models function in the R statistical package MASS was used to adjust gene expression data for age and sex. Residual values deviating by more than three standard deviations from the median were then filtered out. The gene expression profile of patients with high versus low Lp(a) is reported using the *t*-statistic and the Benjamini–Hochberg method to correct for FDR (*p*-value < 0.05).

## 5. Conclusions

Our study revealed new potential mechanisms and Lp(a)-associated proteins that may be involved in the development and progression of CAVS. Some proteins seemed to have a protective effect and could counteract the deleterious effects of Lp(a), whereas other proteins, such as cell-adhesion molecules, may be more deleterious. These proteins could be used as potential new biomarkers or therapeutic targets of CAVS. From our large-scale analysis of the aortic valve transcriptome, we conclude that several mechanisms related to aging, ossification and inflammation may be involved in the development of CAVS in the context of lifelong exposure to high Lp(a) levels.

## Figures and Tables

**Table 1:** Patient characteristics for Lp(a)-isolated particles.

Clinical Characteristics	Control Lp(a) (*n* = 22)	CAVS Lp(a) (*n* = 21)	*p*-Value
Age	61.2 ± 15.1	71.1 ± 4.4	<0.0001
Men, % (*n*)	68.2 (15)	57.1 (12)	0.454
Smoking, %, (*n*)	42.8 (9)	66.7 (14)	0.121
BMI, kg/m^2^	27.6 ± 5.3	30.3 ± 5.8	0.056
Diabetes, %, (*n*)	40.9 (9)	42.9(9)	0.897
Blood pressure, mmHg			
Systolic	129 ± 30.9	133.6 ± 18.7	0.601
Diastolic	77.8 ± 19.5	72.4 ± 9.8	0.148
Cardiovascular disease, %, (*n*)	54.5 (12)	57.1 (12)	0.864
Statin use, %, (*n*)	59.1 (13)	85.7 (18)	0.052
Lipoprotein(a), nmol/L	202.1 ± 84	198.3 ± 81.8	0.759

**Table 2:** List of proteins found to be differentially associated between Lp(a) of subjects with aortic stenosis compared to healthy participants.

Gene	Protein	CAVS/Control Ratio	Limma *p*-Value
LCAT	lecithin–cholesterol acyltransferase	3.4007	0.0029
NCAM1	neural cell adhesion molecule 1	2.9069	0.0070
VASN	Vasorin	3.4835	0.0072
β2GPI	beta-2-glycoprotein 1	0.4060	0.0087
PON3	paraoxonase 3	2.3675	0.0108
SERPING1	serpin family G member 1	2.0398	0.0109
LAMP2	lysosomal-associated membrane protein 2	2.1678	0.0270
IGHM	immunoglobulin heavy constant mu	0.2424	0.0285
IGKV2D-30	immunoglobulin kappa variable 2D-30	0.3026	0.0343
MCAM	melanoma cell adhesion molecule	2.9838	0.0356
MADCAM1	mucosal vascular addressin cell adhesion molecule 1	2.4532	0.0392
CHL1	cell adhesion molecule L1-like	1.7736	0.0427
JCHAIN;IGJ	joining chain of multimeric IgA and IgM	0.5021	0.0479

**Table 3:** Patient characteristics for the explanted valves cohort.

Clinical Characteristics	Low Lp(a) (*n* = 59)	High Lp(a) (*n* = 59)	*p*-Value
Age, years	73.0 ± 6.6	72.8 ± 6.9	0.85
Men, % (*n*)	61.0 (36)	61.0 (36)	1
Smoking, %, (*n*)	5.1 (3)	5.1 (3)	1
BMI, kg/m^2^	29.9 ± 5.1	30.4 ± 6.4	0.61
Diabetes, %, (*n*)	45.8 (27)	37.3 (22)	0.35
Blood pressure, mmHg			
Systolic	134.2 ± 19.8	134.5 ± 20.4	0.71
Diastolic	73.1 ± 9.8	73.1 ± 9.6	0.51
Cardiovascular disease, % (*n*)	57.6 (34)	74.6 (44)	0.051
NYHA severity, %, (*n*)			
1	13.6 (8)	18.6 (11)	
2	47.5 (28)	50.8 (30)	
3	37.3 (22)	27.1 (16)	
4	1.7 (1)	3.4 (2)	
Statin use, %, (*n*)	83.1 (49)	84.7 (49)	1
Lipoprotein(a), nmol/L	29.2 ± 28.6	217.6 ± 93.1	<0.0001

## Data Availability

The microarray gene expression dataset on human aortic valves is available in the Gene Expression Omnibus with accession number GSE102249. The other data available upon reasonable request.

## Supplementary Materials

The following are available online at https://www.mdpi.com/article/10.3390/metabo11070459/s1, Table S1: Characterisation of the proteins found on Lp(a) particles regardless of CAVS status according to their relative abundance, Table S2: Top 10 genes influenced by Lp(a) levels, Table S3: Pathways description and log10(p) from the MCODE algorithm analysis of the top genes identified in the transcriptomic stud. Figure S1: Molecular Complex Detection (MCODE) algorithm network performed for the top genes influenced by Lp(a) levels. MCODE algorithm detects densely connected regions in large protein-protein interaction networks that may represent molecular complexes. MCODE details are represented in Table S3.
