# Supplementary material for: Lipoprotein Proteomics and Aortic Valve Transcriptomics Identify Biological Pathways Linking Lipoprotein(a) Levels to Aortic Stenosis
Source: Metabolites. 2021 Jul 16;11(7):459. doi: 10.3390/metabo11070459 (PMC8307014; doi:10.3390/metabo11070459)
Supplement: Supplementary file 1 [file metabolites-11-00459-s001.zip › metabolites-1293932-sup-v1.pdf]

SUPPLEMENTARY MATERIAL

Lipoprotein Proteomics and Aortic Valve Transcriptomics Identify Biological Pathways Linking Lipoprotein(a) levels to Aortic Stenosis

Raphaëlle Bourgeois[1,2], Jérôme Bourgault[1,2], Audrey-Anne Despres[1,2], Nicolas Perrot[1,2], Jakie Guertin[1,2], Arnaud Girard[1,2], Patricia L. Mitchell[1], Clarisse Gotti[3], Sylvie Bourassa[3], Corey A Scipione[4], Nathalie Gaudreault[1], Michael B Boffa[5], Marlys L. Koschinsky[5], Philippe Pibarot[1,2], Arnaud Droit[3,6], Sébastien Thériault[1,7], Patrick Mathieu[1,8], Yohan Bossé[1,9] and Benoit J Arsenault*[1,2]

1) Centre de recherche de l'Institut universitaire de cardiologie et de pneumologie de Québec, QC, Canada
2) Department of Medicine, Faculty of Medicine, Université Laval, Québec, QC, Canada
3) Proteomics platform of the CHU de Québec, QC, Canada
4) Toronto General Research Institute, University Health Network, ON, Canada
5) Robarts Research Institute, London, ON, Canada
6) Centre de recherche du CHU de Québec, QC, Canada
7) Department of Molecular Biology, Medical Biochemistry and Pathology, Faculty of Medicine, Université Laval, Québec, Québec, Canada.
8) Department of Surgery, Faculty of Medicine, Université Laval, Québec, QC, Canada
9) Department of Molecular Medicine, Faculty of Medicine, Université Laval, Québec, Québec, Canada.

Figure S1. Molecular Complex Detection (MCODE) algorithm network performed for the top genes influenced by Lp(a) levels. MCODE algorithm detects densely connected regions in large protein-protein interaction networks that may represent molecular complexes. MCODE details are represented in supplementary Table 3.

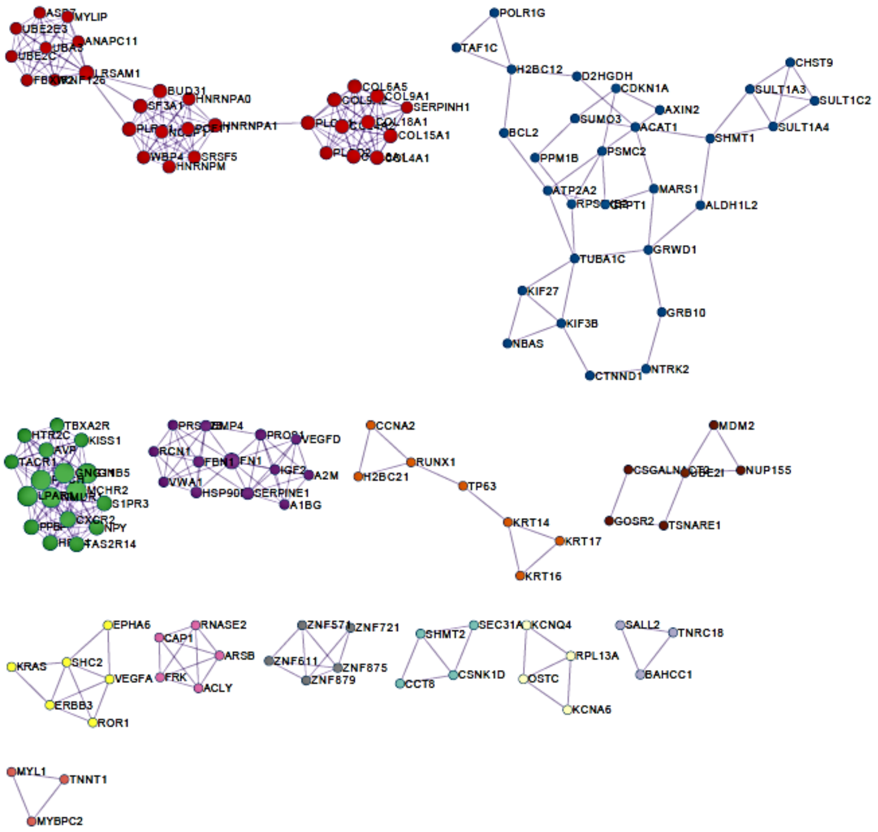

Table S1: Characterisation of the proteins found on Lp(a) particles regardless of CAVS status according to their relative abundance

Gene	Protein	Mean signal
APOB	Apolipoprotein B-100;Apolipoprotein B-48	3,76E+11
LPA	Apolipoprotein(a)	6,85E+10
APOA1	Apolipoprotein A-I	1,40E+10
SERPING1	Plasma protease C1 inhibitor	1,04E+10
APOE	Apolipoprotein E	7,40E+09
APOC3	Apolipoprotein C-III	4,99E+09
A0A0U1RR20		4,53E+09
CLU	Clusterin	2,90E+09
APOA4	Apolipoprotein A-IV	2,16E+09
HP	Haptoglobin	1,93E+09
SERPINA1	Alpha-1-antitrypsin	1,82E+09
PON1	Serum paraoxonase/arylesterase 1	1,54E+09
HBB	Hemoglobin subunit beta	1,22E+09
IGHA1	Ig alpha-1 chain C region	1,15E+09
APOA2	Apolipoprotein A-II	8,66E+08
TTR	Transthyretin	8,58E+08
APOD	Apolipoprotein D	7,62E+08
SERPINA3	Alpha-1-antichymotrypsin	7,14E+08
C3	Complement C3	6,46E+08
KNG1	Kininogen-1	5,64E+08
APOC1	Apolipoprotein C-I	5,16E+08
IGKC	Ig kappa chain C region	5,15E+08
F2	Prothrombin	5,00E+08
HBA1;HBA2	Hemoglobin subunit alpha	4,80E+08
APOC4-APOC2;APOC2	Apolipoprotein C-II	4,69E+08
C4A	Complement C4-A	4,52E+08
VTN	Vitronectin	4,18E+08
APOF	Apolipoprotein F	4,15E+08
ITIH2	Inter-alpha-trypsin inhibitor heavy chain H2	4,01E+08
AMBP	Protein AMBP	3,98E+08
ORM1	Alpha-1-acid glycoprotein 1	3,50E+08
IGHM	Ig mu chain C region	3,49E+08
ITIH3	Inter-alpha-trypsin inhibitor heavy chain H3	3,42E+08
SERPINC1	Antithrombin-III	3,37E+08
ITIH1	Inter-alpha-trypsin inhibitor heavy chain H1	3,16E+08
IGHG1	Ig gamma-1 chain C region	2,97E+08
GP1BA	Platelet glycoprotein Ib alpha chain	2,96E+08
F5	Coagulation factor V	2,81E+08
PCYOX1	Prenylcysteine oxidase 1	2,58E+08

FLNA	Filamin-A	2,45E+08
ACTB;ACTG1	Actin, cytoplasmic 1;Actin, cytoplasmic 1, N-terminally processed	2,26E+08
A2M	Alpha-2-macroglobulin	2,25E+08
PLTP	Phospholipid transfer protein	2,23E+08
IGLL5;IGLC1	Immunoglobulin lambda-like polypeptide 5;Ig lambda-1 chain C regions	2,21E+08
HEG1	Protein HEG homolog 1	2,16E+08
PODXL	Podocalyxin	2,16E+08
GOLM1	Golgi membrane protein 1	2,11E+08
APOM	Apolipoprotein M	2,09E+08
PI16	Peptidase inhibitor 16	1,99E+08
SPARCL1	SPARC-like protein 1	1,97E+08
A0A2R8Y4Z1		1,74E+08
CFH	Complement factor H	1,54E+08
SSC5D	Soluble scavenger receptor cysteine-rich domain-containing protein SSC5D	1,46E+08
FN1	Fibronectin	1,32E+08
PTPRG	Receptor-type tyrosine-protein phosphatase gamma	1,31E+08
CD44	CD44 antigen	1,30E+08
SAA2-SAA4;SAA4	Serum amyloid A-4 protein	1,29E+08
LBP	Lipopolysaccharide-binding protein	1,28E+08
ORM2	Alpha-1-acid glycoprotein 2	1,22E+08
ABI3BP	Target of Nesh-SH3	1,17E+08
AGT	Angiotensinogen	1,07E+08
CENPF	Centromere protein F	1,03E+08
A1BG	Alpha-1B-glycoprotein	9,80E+07
ITGA2B	Integrin alpha-Iib	9,31E+07
HPR	Haptoglobin-related protein	8,92E+07
TF	Serotransferrin	8,80E+07
IGHG2	Ig gamma-2 chain C region	8,68E+07
AHSG	Alpha-2-HS-glycoprotein	7,90E+07
TLN1	Talin-1	7,59E+07
MENT	Protein MENT	7,15E+07
APOC4	Apolipoprotein C-IV	6,87E+07
PLA2G7	Platelet-activating factor acetylhydrolase	6,53E+07
HSPA5	78 kDa glucose-regulated protein	6,30E+07
COL18A1	Collagen alpha-1(XVIII) chain	6,24E+07
PTPRJ	Receptor-type tyrosine-protein phosphatase	5,63E+07
AKAP12	A-kinase anchor protein 12	5,41E+07
C9	Complement component C9	5,35E+07
SERPINF2	Alpha-2-antiplasmin	5,12E+07
YWHAZ	14-3-3 protein zeta/delta	5,09E+07

TNC	Tenascin	4,81E+07
SERPIND1	Heparin cofactor 2	4,53E+07
VCAM1	Vascular cell adhesion protein 1	4,42E+07
ITIH4	Inter-alpha-trypsin inhibitor heavy chain H4	4,35E+07
APOL1	Apolipoprotein L1	4,28E+07
SELL	L-selectin	3,89E+07
CPN2	Carboxypeptidase N subunit 2	3,61E+07
C4BPA	C4b-binding protein alpha chain	3,54E+07
ITGB3	Integrin beta-3	3,45E+07
JCHAIN;IGJ	Immunoglobulin J chain	3,43E+07
C1S	Complement C1s subcomponent	3,34E+07
TNXB	Tenascin-X	3,29E+07
SERPINA1	Alpha-I-antitrypsin	3,21E+07
PTPRZ1	Receptor-type tyrosine-protein phosphatase zeta	3,18E+07
GC	Vitamin D-binding protein	3,15E+07
DCD	Dermcidin	3,13E+07
TTN	Titin	2,99E+07
MUC1	Mucin-1	2,98E+07
C1R	Complement C1r subcomponent	2,93E+07
S100A9	Protein S100-A9	2,87E+07
HRG	Histidine-rich glycoprotein	2,73E+07
VCAN	Versican core protein	2,71E+07
A0A140TA44		2,71E+07
NCAM1	Neural cell adhesion molecule 1	2,70E+07
CP	Ceruloplasmin	2,51E+07
P01619	Ig kappa chain V-III region B6	2,50E+07
CHL1	Neural cell adhesion molecule L1-like protein	2,42E+07
PON3	Serum paraoxonase/lactonase 3	2,31E+07
LCAT	Phosphatidylcholine-sterol acyltransferase	2,30E+07
HPX	Hemopexin	2,26E+07
A0A2Q2TTZ9	Ig kappa chain V-I region	2,18E+07
GPR126	G-protein coupled receptor 126	2,08E+07
PROS1	Vitamin K-dependent protein S	1,95E+07
AFM	Afamin	1,88E+07
NUCB1	Nucleobindin-1	1,88E+07
ITGB1	Integrin beta-1	1,86E+07
S100A8	Protein S100-A8	1,86E+07
CD9	CD9 antigen	1,85E+07
STOM	Erythrocyte band 7 integral membrane protein	1,84E+07
ACTN1	Alpha-actinin-1	1,82E+07
ITGA6	Integrin alpha-6	1,80E+07
GPR116	Probable G-protein coupled receptor 116	1,80E+07
SELP	P-selectin	1,76E+07

LYVE1	Lymphatic vessel endothelial hyaluronic acid receptor 1	1,75E+07
PIP	Prolactin-inducible protein	1,74E+07
VASN	Vasorin	1,71E+07
IGHA2	Ig alpha-2 chain C region	1,71E+07
F10	Coagulation factor X	1,69E+07
VCL	Vinculin	1,68E+07
IGFALS	Insulin-like growth factor-binding protein complex acid labile subunit	1,68E+07
CD34	Hematopoietic progenitor cell antigen CD34	1,59E+07
MADCAM1	Mucosal addressin cell adhesion molecule 1	1,57E+07
MCAM	Cell surface glycoprotein MUC18	1,52E+07
PTPRC	Receptor-type tyrosine-protein phosphatase C	1,47E+07
MUC20	Mucin-20	1,43E+07
IGHG3	Ig gamma-3 chain C region	1,39E+07
TFPI	Tissue factor pathway inhibitor	1,35E+07
LAMP2	Lysosome-associated membrane glycoprotein 2	1,30E+07
SYNE1	Nesprin-1	1,29E+07
CPN1	Carboxypeptidase N catalytic chain	1,29E+07
SEPP1	Selenoprotein P	1,27E+07
CD248	Endosialin	1,25E+07
HLA-A	HLA class I histocompatibility antigen, A-24 alpha chain	1,23E+07
F13A1	Coagulation factor XIII A chain	1,20E+07
SPN	Leukosialin	1,17E+07
PGLYRP2	N-acetylmuramoyl-L-alanine amidase	1,12E+07
C5	Complement C5	1,12E+07
FERMT3	Fermitin family homolog 3	1,10E+07
SERPINA10	Protein Z-dependent protease inhibitor	1,08E+07
UBC;UBB;RPS27A;UBA52	Ubiquitin; Polyubiquitin-C; Ubiquitin-40S ribosomal protein S27a; Ubiquitin-60S ribosomal protein L40	9,51E+06
APCS	Serum amyloid P-component	9,16E+06
CD36	Platelet glycoprotein 4	9,14E+06
P4HB	Protein disulfide-isomerase	9,14E+06
IGLV3-9	Ig lambda chain V-III region LOI	8,79E+06
GPLD1	Phosphatidylinositol-glycan-specific phospholipase D	8,77E+06
FBLN1	Fibulin-1	8,40E+06
AZGP1	Zinc-alpha-2-glycoprotein	8,12E+06
C1RL	Complement C1r subcomponent-like protein	7,80E+06
IGKV2D-30	Ig kappa chain V-II region RPMI 6410	7,34E+06
BASP1	Brain acid soluble protein 1	7,28E+06
SERPINA6	Corticosteroid-binding globulin	6,88E+06

CETP	Cholesteryl ester transfer protein	6,79E+06
APOH	Beta-2-glycoprotein 1	6,66E+06
ANPEP	Aminopeptidase N	6,57E+06
B2M	Beta-2-microglobulin	6,50E+06
MXRA5	Matrix-remodeling-associated protein 5	5,56E+06
LGALS3BP	Galectin-3-binding protein	3,78E+06
MEGF9	Multiple epidermal growth factor-like domains protein 9	3,61E+06
PARVB	Beta-parvin	3,36E+06
CAMP	Cathelicidin antimicrobial peptide	3,34E+06
MANSC1	MANSC domain-containing protein 1	2,96E+06
SUSD5	Sushi domain-containing protein 5	2,65E+06
CRTAC1	Cartilage acidic protein 1	2,46E+06

Table S2: Top 10 genes influenced by Lp(a) levels

LogFC high minus low Lp(a)	Average Expression	T	p-value	p-value FDR	B	Gene	Chromosome
**-0,1081**	6,5926	-4,9045	2,943E-06	0,0696	4,342	SERPINB9	chr6
**-0,1390**	7,0563	-4,3916	2,423E-05	0,2594	2,442	ADAMTSL5	chr19
**-0,1614**	7,0877	-4,3112	3,327E-05	0,2594	2,157	SGTB	chr5
**0,1756**	6,6305	4,2696	3,914E-05	0,2594	2,011	NANOS1	chr10
**-0,1233**	8,1592	-4,2251	4,652E-05	0,2594	1,856	PVR	chr19
**0,0988**	6,0877	4,1890	5,347E-05	0,2594	1,732	PNMT	chr17
**-0,0834**	6,1023	-4,1477	6,262E-05	0,2594	1,590	VWDE	chr7
**-0,1233**	10,6033	-4,1241	6,854E-05	0,2594	1,509	EHD1	chr11
**0,0929**	6,3793	4,0159	1,031E-04	0,3142	1,145	ZFP62	chr5
**-0,1371**	6,9186	-3,9799	1,179E-04	0,3142	1,025	RASD2	chr22

Table S3: Pathways description and log10(p) from the MCODE algorithm analysis of the top genes identified in the transcriptomic stud.

Color	MCODE	GO	Description	Log10(P)
	MCODE_1	R-HSA-1650814	Collagen biosynthesis and modifying enzymes	−21.5
	MCODE_1	R-HSA-1474290	Collagen formation	−20.0
	MCODE_1	R-HSA-8948216	Collagen chain trimerization	−16.0
	MCODE_2	R-HSA-156584	Cytosolic sulfonation of small molecules	−5.7
	MCODE_2	GO:0051923	sulfation	−5.7
	MCODE_2	GO:0034035	purine ribonucleoside bisphosphate metabolic process	−5.7
	MCODE_3	R-HSA-500792	GPCR ligand binding	−30.4
	MCODE_3	R-HSA-373076	Class A/1 (Rhodopsin-like receptors)	−24.2
	MCODE_3	R-HSA-416476	G alpha (q) signalling events	−19.3
	MCODE_4	R-HSA-381426	Regulation of Insulin-like Growth Factor (IGF) transport and uptake by Insulin-like Growth Factor Binding Proteins (IGFBPs)	−15.8
	MCODE_4	R-HSA-8957275	Post-translational protein phosphorylation	−13.8
	MCODE_4	GO:0002576	platelet degranulation	−13.2
	MCODE_5	GO:0030216	keratinocyte differentiation	−8.5
	MCODE_5	GO:0042303	molting cycle	−8.2
	MCODE_5	GO:0042633	hair cycle	−8.2
	MCODE_6	GO:0033674	positive regulation of kinase activity	−10.0
	MCODE_6	GO:0051347	positive regulation of transferase activity	−9.7
	MCODE_6	ko01521	EGFR tyrosine kinase inhibitor resistance	−9.1
	MCODE_7	R-HSA-3232142	SUMOylation of ubiquitinylation proteins	−7.3
	MCODE_7	GO:0016925	protein sumoylation	−6.3
	MCODE_7	R-HSA-3108232	SUMO E3 ligases SUMOylate target proteins	−5.3

	MCODE_8	R-HSA-6798695	Neutrophil degranulation	−8.8
	MCODE_8	GO:0043312	neutrophil degranulation	−8.8
	MCODE_8	GO:0002283	neutrophil activation involved in immune response	−8.8
	MCODE_9	hsa05168	Herpes simplex virus 1 infection	−8.5
	MCODE_13	R-HSA-390522	Striated Muscle Contraction	−8.7
	MCODE_13	WP383	Striated Muscle Contraction Pathway	−8.6
	MCODE_13	GO:0033275	actin-myosin filament sliding	−8.6
